# Cyclosporine A causes gingival overgrowth via reduced G_1_ cell cycle arrest in gingival fibroblasts

**DOI:** 10.1371/journal.pone.0309189

**Published:** 2024-12-20

**Authors:** Reiri Takeuchi, Takatoshi Nomura, Manabu Yaguchi, Noriko Kuwahara, Yuta Amino, Chieko Taguchi, Itaru Suzuki, Haruka Suzuki, Teruaki Nagashima, Kazumune Arikawa, Yuichiro Okada, Takato Nomoto, Koichi Hiratsuka

**Affiliations:** 1 Department of Biochemistry and Molecular Biology, Nihon University School of Dentistry at Matsudo, Matsudo, Chiba, Japan; 2 Department of Special Needs Dentistry, Nihon University School of Dentistry at Matsudo, Matsudo, Chiba, Japan; 3 Department of Oral Implantology, Nihon University School of Dentistry at Matsudo, Matsudo, Chiba, Japan; 4 Department of Community Oral Health, Nihon University School of Dentistry at Matsudo, Matsudo, Chiba, Japan; 5 Department of Community Oral Health, Nihon University Graduate School of Dentistry at Matsudo, Matsudo, Chiba, Japan; 6 Department of Histology, Nihon University School of Dentistry at Matsudo, Matsudo, Chiba, Japan; Fujita Health University, JAPAN

## Abstract

Gingival overgrowth caused by cyclosporine A is due to increased fibroblast proliferation in gingival tissues. Cell cycle system balances proliferation and anti-proliferation of gingival fibroblasts and plays a role in the maintenance of its population in gingival tissues. When cells detect and respond to abnormalities (e.g. DNA damage), cell cycle progression is arrested in the G_1_ phase until the completion of damage restoration. In this study, we investigated the effects of cyclosporine A on G_1_ cell cycle arrest and on its regulators in gingival fibroblasts to clarify the mechanism of cyclosporine A-induced gingival overgrowth. Human gingival fibroblasts from healthy donors were cultured to semi-confluence and were then treated with or without 200 ng/mL (166 nM) cyclosporine A in D-MEM with 2% fetal bovine serum. Cell proliferation was assessed by counting total cell numbers. The distribution of cell cycle phases was assessed using flow cytometric analysis. The levels of mRNA and protein expression for cell cycle regulators were quantified using reverse transcription-quantitative PCR and western blot analysis, respectively. Treatment with cyclosporine A markedly increased cell proliferation, inhibited G_1_ cell cycle arrest, significantly increased *CDC25A* and *CYCLIN E1* mRNA expression levels, significantly decreased *P21*, *SMAD3* and *SMAD4* mRNA expression levels, significantly upregulated the protein expression levels of CDC25A, CYCLIN E1, pCDK2 and pRB1 and significantly downregulated the protein expression levels of P21, SMAD3 and SMAD4. Treatment with cyclosporine A also increased *MYC* and *ATM* mRNA expression levels and decreased *CDK2*, *ATR*, *P27*, *P53* and *RB1* mRNA expression levels but not significantly. These results demonstrate that cyclosporine A causes gingival overgrowth due to the following mechanism in gingival fibroblasts: cyclosporine A increases levels of phospho-CDK2 and CYCLIN E1 by upregulating CDC25A and downregulating P21 with the downregulation of SMAD3 and SMAD4, which results in the inhibition of G_1_ cell cycle arrest.

## Introduction

Drug-induced gingival overgrowth is caused by a side-effect of drugs such as cyclosporine A (CsA, an immunosuppressant), phenytoin (an antiepileptic) and nifedipine (a calcium channel blocker) [1–4]. This disease is also referred to as “drug-induced gingival enlargement” or was previously known as “drug-induced gingival hyperplasia” [1]. The gingival overgrowth clinically causes an enlargement of the size of the gingiva however the intended target organ of these drugs is not the gingival tissue [1, 5]. The overgrowth of the gingiva impedes proper dental hygiene, and not only disrupts normal mastication and causes painful chewing and eating but also leads to cosmetic damage that may contribute to psychological distress [1, 4, 6–8].

CsA is most frequently prescribed for immunosuppression after an organ transplant, and the prevalence of CsA-induced gingival overgrowth in cross-sectional studies is estimated to be 50–80% [1, 9]. Many organ transplants (157,494 cases) were performed in 91 countries in 2022 [10].

The following various processes cause the development of fibrosis in gingival tissues exposed to CsA: increase in the cell proliferation, decrease in the cell apoptosis, and accumulation of collagen in gingival fibroblasts [9]. CsA induces gingival overgrowth through promoting the proliferation of gingival fibroblasts [11, 12]. The gingival fibroblast is principal cell that takes responsibility for the maintenance and repair of gingival connective tissue [13]. The pathogenesis of gingival overgrowth caused by CsA has been studied using an *in vitro* model and several researches have investigated the effects of CsA on cultured gingival fibroblasts [12, 14].

Cell cycle system balances proliferation and anti-proliferation of gingival fibroblasts and plays a role in the maintenance of its population in gingival tissues [15, 16]. There are four different cell cycle phases: S phase for DNA replication, M phase for cell division, and G_1_ and G_2_ phases that separate the S and M phases [15, 16]. Additionally, there are the checkpoint mechanisms, the G_1_ checkpoint, the G_2_ checkpoint, etc, to control the cell cycle progression [15, 16]. When cells detect and respond to abnormalities (e.g. DNA damage), cell cycle progression is arrested at the G_1_ checkpoint until the completion of damage restoration [15, 16]. The mechanism underlying gingival overgrowth by phenytoin has been reported to be associated with reduced G_1_ cell cycle arrest in gingival fibroblasts [17], however, the mechanism involved in the gingival overgrowth induced by CsA is unclear to date and required further investigation.

In this study, we investigated the effects of CsA on G_1_ cell cycle arrest and on its regulators in gingival fibroblasts, to clarify the mechanism of CsA-induced gingival overgrowth. The results show that CsA modulates the expression of proteins regulating the transition to S phase from the G_1_ phase in gingival fibroblasts, which leads to the inhibition of G_1_ cell cycle arrest.

## Materials and methods

### Cell line and culture

The methods used in this study are based on previously published reports [4, 5, 15, 17, 18]. Primary gingival fibroblasts derived from the healthy donors used in this study were obtained from ScienCell^TM^ Research Laboratories (cat. no. 2620, San Diego, CA, USA). Those gingival fibroblasts had been cryopreserved at passage one and were delivered frozen. Cells were cultured according to manufacturer’s instructions in Dulbecco’s modified Eagle medium (High Glucose) with L-Glutamine and Phenol Red (D-MEM, FUJIFILM Wako Pure Chemical Corp., Osaka, Japan) supplemented with 10% fetal bovine serum, 50 units/ml penicillin and 50 μg/ml streptomycin (Gibco, Thermo Fisher Scientific, Inc., Waltham, MA, USA). Cells were grown under an atmosphere of 5% CO_2_/95% air and were maintained at 37°C until they reached semi-confluence after which they were routinely passaged using 0.05 w/v% Trypsin-0.53 mmol/L EDTA-4Na Solution with Phenol Red (FUJIFILM Wako Pure Chemical Corp.). Gingival fibroblasts were used between passages 6 and 9 for these experiments. CsA (cat. no. 031–24931) was purchased from FUJIFILM Wako Pure Chemical Corp. (Osaka, Japan). The concentration of CsA used in this study was decided according to the results of a previous study [19] as follows: 200 ng/mL (166 nM) CsA significantly induced the proliferation of normal gingival fibroblasts compared with the untreated control.

### Determination of cell proliferation

Cell proliferation was determined by counting total cell numbers with Trypan Blue dye exclusion using a blood corpuscle counting chamber (Burker-Turk deep 1/10 mm, ERMA Inc., Tokyo, Japan), and cellular morphology was examined using an optical microscope (Nikon TE300 Inverted Tissue Culture Microscope, Tokyo, Japan; magnification, 40×). After cells were cultured until they reached semi-confluence, they were treated with or without 200 ng/mL (166 nM) CsA in D-MEM with 2% fetal bovine serum for 24 or 48 h. The methods used in this study are based on a previously published report [15].

### Investigation of cell cycle arrest

Cell cycle arrest was investigated by flow cytometric analysis using a CycleTEST^TM^ plus DNA Reagent Kit (Becton Dickinson and Company, Franklin Lakes, NJ, USA). Semi-confluent cells were treated with or without 200 ng/mL (166 nM) CsA in D-MEM with 2% fetal bovine serum for 24 h to induce cell cycle arrest at the G_0_/G_1_ phase. After treatment, cells were harvested by trypsinization, washed three times with Buffer Solution, and then treated with Solution A (trypsin buffer), Solution B (trypsin inhibitor and RNase buffer) and Solution C (propidium iodide stain solution) in accordance with the manufacturer’s instructions. Two x 10^5^ cells in each sample were measured using a FACSCalibur™ Flow Cytometer (BD Biosciences). The percentage of cells in the G_0_/G_1_, S and G_2_/M phases of the cell cycle were determined using BD CellQuest Pro Software (version 3.1, BD Biosciences). The methods used in this study are based on previously published reports [4, 5, 15, 17].

### Analysis of mRNA expression

mRNA expression levels were analyzed using RNA isolation and reverse transcription-quantitative PCR (RT-qPCR). After cells were cultured until they reached semi-confluence, they were treated with or without 200 ng/mL (166 nM) CsA in D-MEM with 2% fetal bovine serum for 12 h, after which total RNA was immediately extracted from the cells using a RNeasy Mini Kit (QIAGEN, Tokyo, Japan). The concentration and purity of each total RNA was assessed by a standard spectrophotometric method. One μg of each total RNA was then reverse-transcribed using a PrimeScript™ RT reagent Kit (TAKARA BIO INC., Shiga, Japan). The cDNAs were analyzed by qPCR using an Eco™ Real-Time PCR System (Illumina, Inc., San Diego, CA, USA) with KAPA SYBR® FAST qPCR Master Mix Kit (KAPA BIOSYSTEMS Inc., Wilmington, MA, USA). The following thermocycling conditions were used for qPCR: Enzyme activation at 95˚C for 30 sec, followed by 45 cycles of denaturation at 95˚C for 5 sec and annealing and extension at 60˚C for 20 sec. A Perfect Real Time Support System (TAKARA BIO INC.) was used to synthesize the following PCR primers: ATM serine/threonine kinase (*ATM*); ATR serine/threonine kinase (*ATR*); cell division cycle 25A (*CDC25A*); cyclin dependent kinase 2 (*CDK2*); E-type cyclin (*CYCLIN E1*); MYC proto-oncogene bHLH transcription factor (*MYC*); cyclin dependent kinase inhibitor 1A (*P21*); cyclin dependent kinase inhibitor 1B (*P27*); tumor protein p53 (*P53*); RB transcriptional corepressor 1 (*RB1*); SMAD family member 3 (*SMAD3*); SMAD family member 4 (*SMAD4*); and glyceraldehyde-3-phosphate dehydrogenase (*GAPDH*). These primers were synthesized by Custom DNA Oligos (Merck KGaA, Darmstadt, Germany) using the nucleotide database of the National Center for Biotechnology Information (National Library of Medicine, National Institutes of Health, Bethesda, MD, USA) and Primer3Plus. Primer sequences used are listed in Table 1. Relative quantification was calculated using the 2‑ΔΔCq method [20]. After normalization to GAPDH, RNA ratios in treated vs control cultures were determined. The methods used in this study are based on previously published reports [4, 5].

**Table 1:** Primers used for reverse transcription-quantitative PCR.

Gene symbol	Sequence (5’-3’)	Product size, bp	GenBank accession no.
*ATM*	F: TGAGTGGCAGCTGGAAGAAG	124	NM_001351834.2
	R: TAGGCTGGGATTGTTCGCTG		
*ATR*	F: GTCACCACCAGACAGCCTAC	220	NM_001184.4
	R: CGGCCCACTAGTAGCATAGC		
*CDC25A*	F: CAAGGGTGCAGTGAACTTGC	95	NM_001789.3
	R: CAACAATGACACGCTTGCCA		
*CDK2*	F: TTCCAGGATGTGACCAAGCC	180	NM_001798.5
	R: GGCTGGCCAAGACTAGAAGG		
*CYCLIN E1*	F: TGTCCTGGATGTTGACTGCC	267	NM_001238.4
	R: CGGGCTTTGTCCAGCAAATC		
*MYC*	F: CATCAGCACAACTACGCAGC	169	NM_002467.6
	R: CGTTGTGTGTTCGCCTCTTG		
*P21*	F: TCTAGGAGGGAGACACTGGC	109	NM_000389.5
	R: TGTCTGACTCCTTGTTCCGC		
*P27*	F: AGGGGCGCTTTGTTTTGTTC	222	NM_004064.5
	R: TGCAGTGCTTCTCCAAGTCC		
*P53*	F: CCTGAGGTTGGCTCTGACTG	241	NM_001276760.3
	R: CACGCACCTCAAAGCTGTTC		
*RB1*	F: ATGTCTTTATTGGCGTGCGC	123	NM_000321.3
	R: AGAGCCATGCAAGGGATTCC		
*SMAD3*	F: TCTGAGAGGGCCAAATGCTG	86	NM_005902.4
	R: CAGGGGGCTTCCTGTGTAAG		
*SMAD4*	F: TGGTAGAGGCCAGCTTTGTG	103	NM_005359.6
	R: TCAATCCAAGCCCGTGAGTC		
*GAPDH*	F: GCACCGTCAAGGCTGAGAAC	138	NM_002046.5
	R: TGGTGAAGACGCCAGTGGA		

*ATM*, ATM serine/threonine kinase; *ATR*, ATR serine/threonine kinase; *CDC25A*, cell division cycle 25A; *CDK2*, cyclin dependent kinase 2; *CYCLIN E1*, E-type cyclin; *MYC*, MYC proto-oncogene, bHLH transcription factor; *P21*, cyclin dependent kinase inhibitor 1A; *P27*, cyclin dependent kinase inhibitor 1B; *P53*, tumor protein p53; *RB1*, RB transcriptional corepressor 1; *SMAD3*, SMAD family member 3; *SMAD4*, SMAD family member 4; *GAPDH*, glyceraldehyde-3-phosphate dehydrogenase.

### Analysis of protein expression

The expression levels of proteins were analyzed by western blotting. After cells were cultured until they reached semi-confluence, they were treated with or without 200 ng/mL (166 nM) CsA in D-MEM with 2% fetal bovine serum for 24 h. The cells were then lysed using β-ME Sample Treatment for Tris SDS (COSMO BIO Co., LTD, Tokyo, Japan) after washing with PBS at 37°C. A TaKaRa Bradford Protein Assay Kit (TAKARA BIO INC.) was used to determine protein concentrations. After protein components (10 μg/lane) were separated via SDS-PAGE with Running Buffer Solution for SDS-PAGE (NACALAI TESQUE, INC., Kyoto, Japan), the proteins were transferred to PVDF membranes. The membranes were blocked for 30 min at room temperature with Bullet Blocking One for Western Blotting (NACALAI TESQUE), after which they were probed at room temperature for 1 h with primary antibodies against CDC25A, CYCLIN E1, pCDK2, pRB1, P21, SMAD3, SMAD4 and β-Actin. The membranes were washed three times with Tris Buffered Saline with 0.05%-Detergent (NACALAI TESQUE) for 5 min at room temperature, and were then incubated with the secondary antibody at room temperature for 45 min. All primary and secondary antibodies were diluted to 1:1,000 and 1:10,000, respectively, using Can Get Signal^®^ Immunoreaction Enhancer Solution (TOYOBO CO., LTD., Osaka, Japan). After washing, the blots were detected using Chemi-Lumi One Super (NACALAI TESQUE) and a ChemiDocTM MP Imaging System (Bio-Rad Laboratories, Inc., Hercules, CA, USA). The densities of western blot bands were measured using ImageJ (1.53t; Java 1.8.0_345 [64-bit]). Primary antibodies against pCDK2 (cat. no. #2561), pRB1 (cat. no. #9308), P21 (cat. no. #2947), SMAD3 (cat. no. #9523), SMAD4 (cat. no. #46535) and β-Actin (cat. no. #4967), as well as anti-rabbit HRP-conjugated IgG, and CYCLIN E1 (anti-mouse IgG, HRP-linked antibody; cat. no. #4129) were purchased from Cell Signaling Technology, Inc. (Danvers, MA, USA). The rabbit anti-CDC25A antibody (cat. no. 55031-1-AP) was purchased from Proteintech Group, Inc (Rosemont, IL, USA). The secondary HRP-linked antibodies against rabbit IgG (cat. no. #65–6120) and mouse IgG (cat. no. NA931V) were purchased from Invitrogen, Thermo Fisher Scientific Inc. (Waltham, MA, USA) and GE Healthcare UK Limited Amersham Place (Buckinghamshire, UK), respectively. The methods used in this study are based on previously published reports [4, 5].

### Statistical analysis

All data are presented as means ± standard error of the mean (SEM). Statistical analysis was performed using Welch’s t-test. *P* values < 0.05 are considered to indicate a statistically significant difference.

## Results

### Effect of CsA on the proliferation of gingival fibroblasts

**Fig 1:**
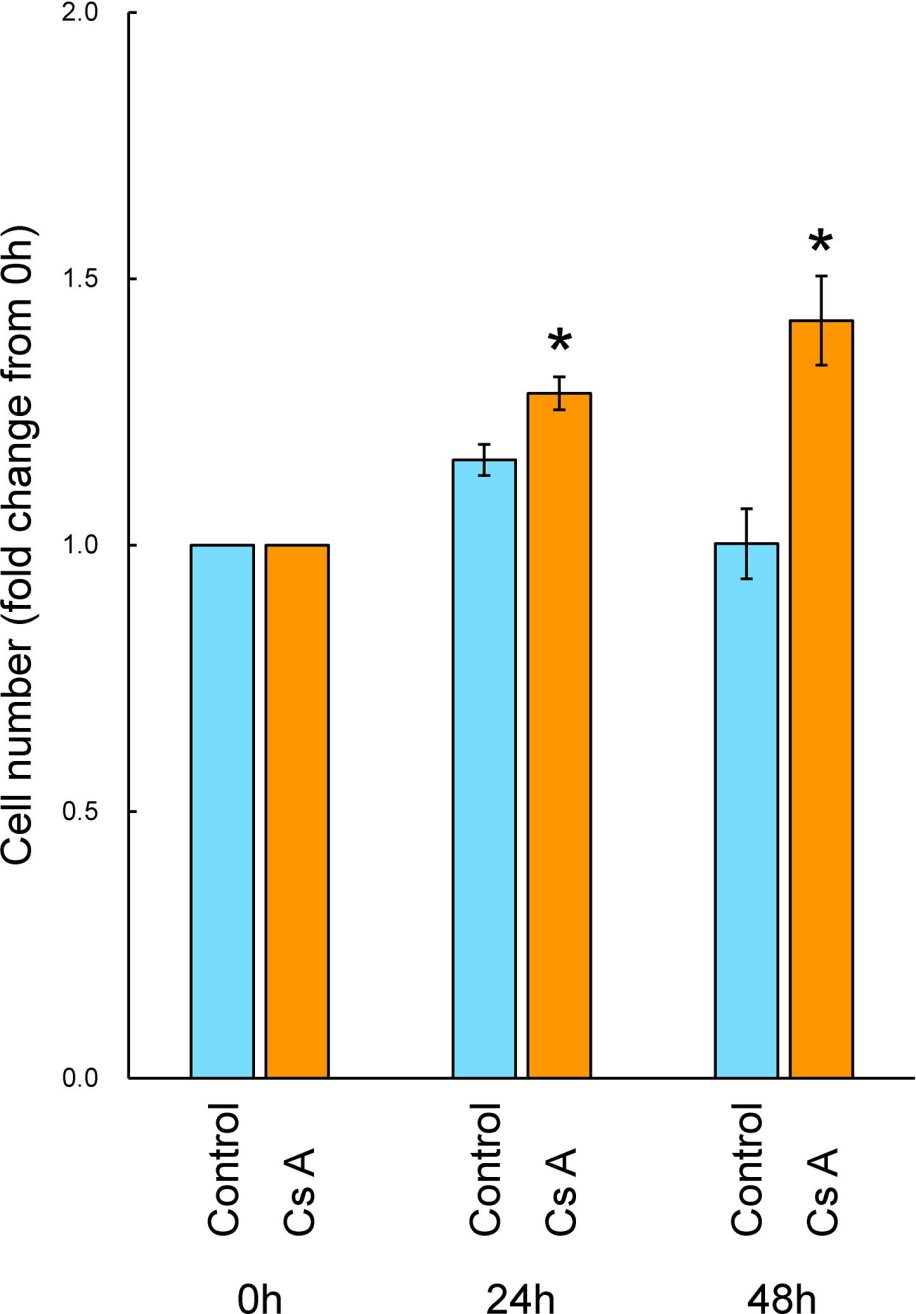
Cell proliferation of gingival fibroblasts cultured in D-MEM with 2% serum in the presence or absence of CsA. After semiconfluent cells were treated with or without (control) 200 ng/mL (166 nM) CsA in D-MEM containing 2% serum for 24 and 48 h, the total cell numbers were measured with Trypan Blue dye exclusion using a blood corpuscle counting chamber and an optical microscope. After normalization to 0 h, the fold change compared with the control was determined. Data are presented as means ± SEM. *P<0.05 compared with the control using Welch’s t-test (n = 4).

Cell proliferation in gingival fibroblasts was evaluated following treatment with or without CsA. A time-dependent increase in the number of cells was shown in gingival fibroblasts treated with CsA. An increase or a decrease in the number of cells were shown in the untreated control cells cultured at 24 h or 48 h, respectively. In contrast, the numbers of gingival fibroblasts exposed to CsA were significantly greater than those of the control at 24 h (1.1-fold) and at 48 h (1.4-fold) (Fig 1).

### Effect of CsA on cell cycle arrest in gingival fibroblasts

**Fig 2:**
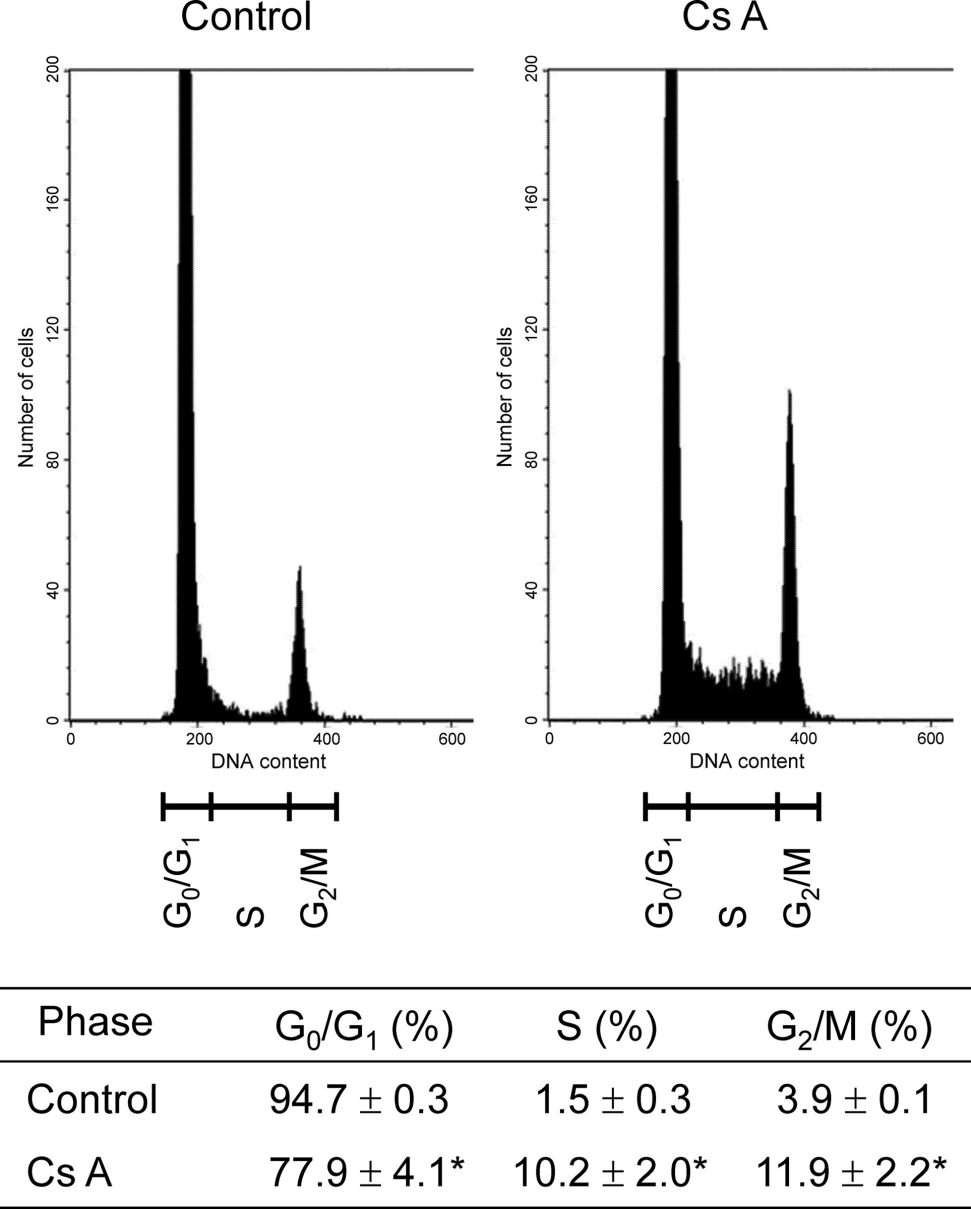
The distribution of cell cycle phases of gingival fibroblasts cultured in the presence or absence of CsA. Semiconfluent cells were incubated in D-MEM containing 2% serum with or without (control) 200 ng/mL (166 nM) CsA for 24 h and then subjected to flow cytometric analysis. A representative histogram from four independent experiments is shown. Detailed values of cell cycle parameters are shown in the Table at the bottom. Data are presented as means ± SEM. *P<0.05 compared with the control using Welch’s t-test (n = 4).

We analyzed the distribution of cell cycle phases (G_0_/G_1_, S and G_2_/M) in gingival fibroblasts that were either treated with CsA or left untreated. Treatment with CsA significantly decreased or increased the cell numbers in the G_0_/G_1_ phase or in the S and G_2_/M phases, respectively, compared to the untreated control (Fig 2).

### Effect of CsA on mRNA expression in gingival fibroblasts

**Fig 3:**
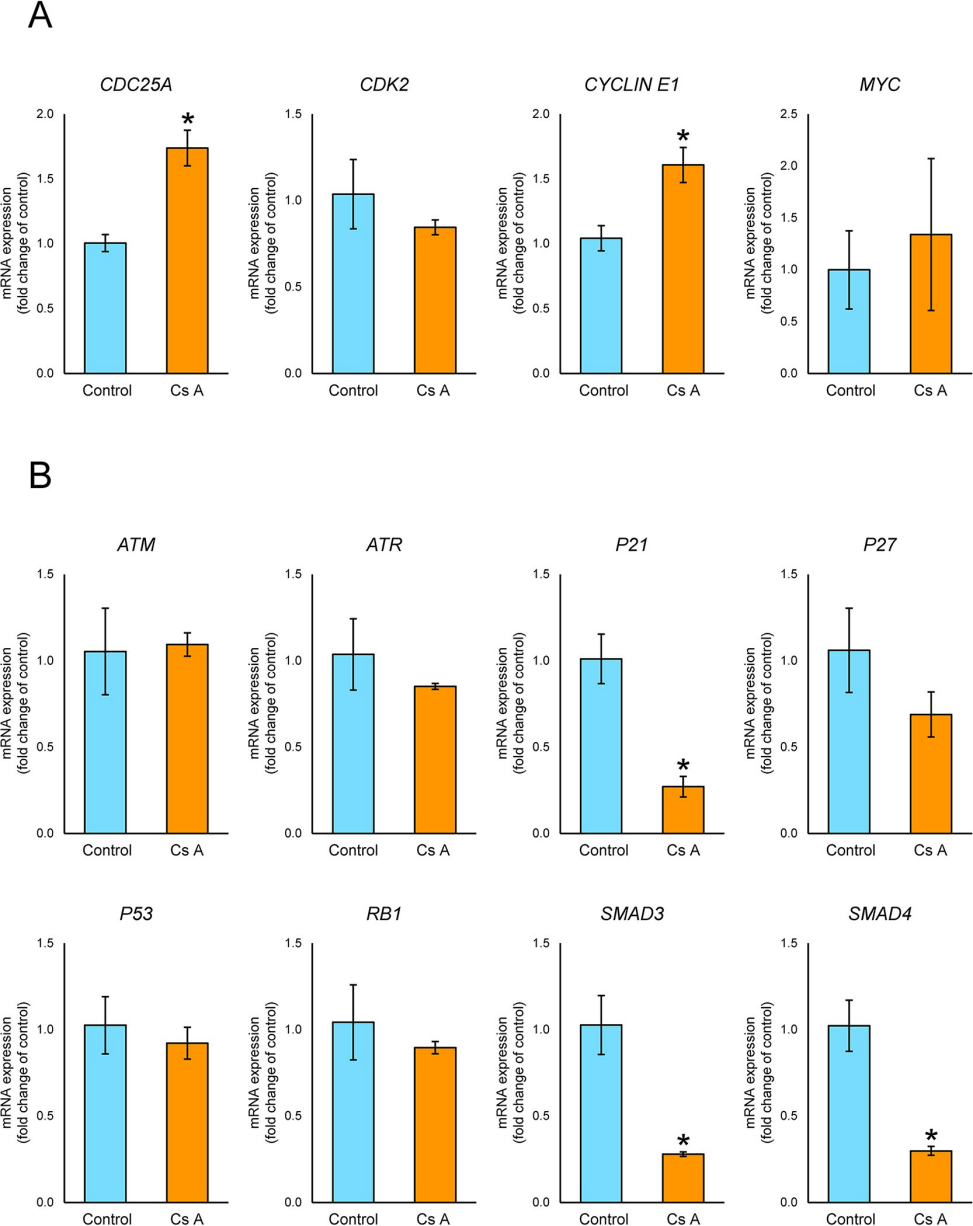
mRNA expression levels of cell cycle regulators for G_1_-S transition in gingival fibroblasts treated with or without CsA. Semiconfluent cells were incubated in D-MEM containing 2% serum with or without (control) 200 ng/mL (166 nM) CsA for 12 h, after which reverse transcription-quantitative PCR analysis was performed. Relative quantification was performed using the 2‑ΔΔCq method. After normalization to GAPDH, RNA ratios in treated vs. control cultures were determined. Data are presented as means ± SEM. *P<0.05 compared with the control using Welch’s t-test (n = 3). (A) G_1_-S transition-promoted genes (*CDC25A*, cell division cycle 25A; *CDK2*, cyclin dependent kinase 2; *CYCLIN E1*, E-type cyclin; *MYC*, MYC proto-oncogene, bHLH transcription factor). (B) G_1_ arrest-induced genes (*ATM*, ATM serine/threonine kinase; *ATR*, ATR serine/threonine kinase; *P21*, cyclin dependent kinase inhibitor 1A; *P27*, cyclin dependent kinase inhibitor 1B; *P53*, tumor protein p53; *RB1*, RB transcriptional corepressor 1; *SMAD3*, SMAD family member 3; *SMAD4*, SMAD family member 4).

We assessed the effects of CsA on the mRNA expression levels of regulators involved in G_1_ cell cycle arrest (*ATM*, *ATR*, *CDC25A*, *CDK2*, *CYCLIN E1*, *MYC*, *P21*, *P27*, *P53*, *RB1*, *SMAD3*, *SMAD4*) in gingival fibroblasts using qPCR. Treatment of gingival fibroblasts with CsA significantly upregulated the mRNA expression levels of *CDC25A* (1.7-fold) and *CYCLIN E1* (1.5-fold), while significantly downregulating the levels of *P21* (0.3-fold), *SMAD3* (0.3-fold) and *SMAD4* (0.3-fold) mRNA. CsA treatment also led to a slight increase in *MYC* (1.3-fold) mRNA expression levels and a slight decrease in *CDK2* (0.8-fold), *ATR* (0.9-fold) and *P27* (0.6-fold) mRNA expression levels but not at a significant level. The mRNA expression levels of *ATM*, *P53*, *RB1* in the cells treated with CsA were almost the same as control (Fig 3).

### Effect of CsA on protein expression in gingival fibroblasts

**Fig 4:**
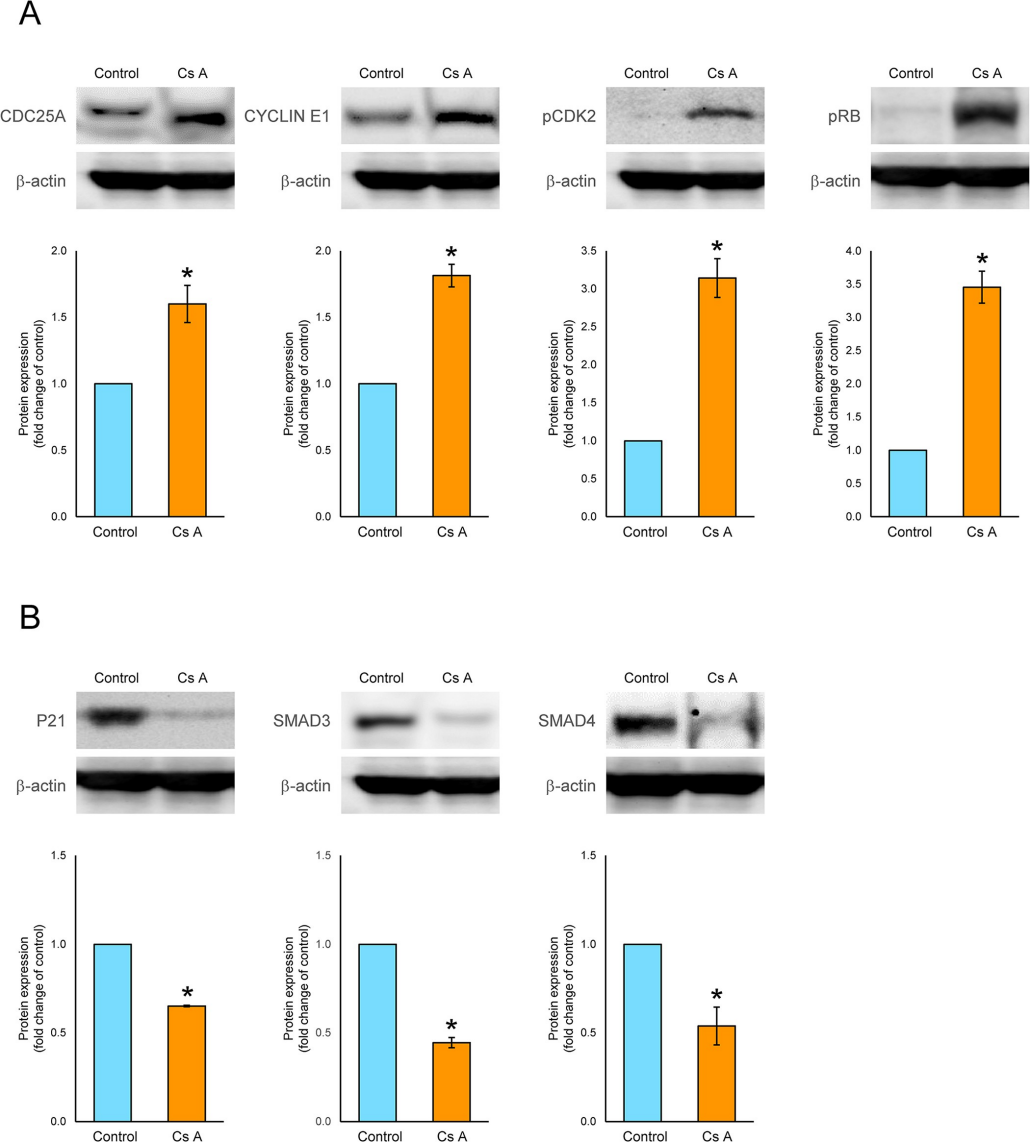
Protein expression levels of cell cycle regulators for G_1_-S transition in gingival fibroblasts treated with or without CsA. Semiconfluent cells were incubated in D-MEM containing 2% serum with or without (control) 200 ng/mL (166 nM) CsA for 24 h and were then assessed using western blotting, after which the fold change compared with the control was determined. The band images shown are representative of results from three independent experiments. Data are presented as means ± SEM. *P<0.05 compared with the control using Welch’s t-test (n = 3). (A) G_1_-S transition-promoted proteins (CDC25A, cell division cycle 25A; CYCLIN E1, E-type cyclin; pCDK2, phospho-cyclin dependent kinase 2; RB1, RB transcriptional corepressor 1). (B) G_1_ arrest-induced proteins (P21, cyclin dependent kinase inhibitor 1A; SMAD3, SMAD family member 3; SMAD4, SMAD family member 4).

We assessed the effects of CsA on the expression of regulatory proteins for G_1_ cell cycle arrest (CDC25A, CYCLIN E1, pCDK2, pRB1, P21, SMAD3 and SMAD4) in gingival fibroblasts using western blotting. Treatment of gingival fibroblasts with CsA significantly elevated the levels of protein expression of CDC25A (1.6-fold), CYCLIN E1 (1.8-fold), pCDK2 (3.1-fold) and pRB1 (3.5-fold) and significantly decreased the protein expression levels of P21 (0.7-fold), SMAD3 (0.4-fold) and SMAD4 (0.5-fold) compared to the protein expression levels observed in untreated control cells (Fig 4).

## Discussion

This study aimed to clarify the mechanism of gingival overgrowth that is induced by CsA. We investigated the effects of CsA on G_1_ cell cycle arrest and on its regulators in gingival fibroblasts and found that CsA inhibited G_1_ cell cycle arrest through an increase in inducers and a decrease in inhibitors of the transition into S phase from the G_1_ phase in gingival fibroblasts.

In this study, we cultured gingival fibroblasts in D-MEM supplemented with 2% fetal bovine serum to induce G_1_ cell cycle arrest and decrease cell proliferation. Meanwhile, the cells were treated with CsA in D-MEM containing 2% serum to assess the effects of CsA on their response.

Some studies have indicated that there is no correlation between the blood levels or oral dosage of CsA and the incidence of gingival overgrowth [21–23]. One study found that the serum concentration of CsA in patients receiving immunosuppressive therapy based on cyclosporine A was 128.82 ± 59.60 ng/mL [23]. Additionally, a previous *in vitro* study utilized a concentration of 200 ng/mL (166 nM) of cyclosporine A to examine its effects on the proliferation of cultured gingival fibroblasts [19]. We referenced these studies to determine the concentration of CsA employed in this research.

CsA induces an increase in the cell proliferation and a decrease in the cell apoptosis in gingival fibroblasts, and this drug causes gingival overgrowth [14, 19]. The development of drug-induced gingival overgrowth is associated with the interaction between these facts and the inflammation in gingiva [24]. In cells, the progression and the arrest of cell cycle controls cell proliferation. We counted the total numbers of gingival fibroblasts exposed to CsA to assess cell proliferation, and the results showed that CsA increased the number of fibroblasts. The average proliferation ratios of untreated control cells compared to the 0-hour measurement remained stable, showing values of 1.03-fold at 12 hours (data not shown), 1.16-fold at 24 hours and 1.00-fold at 48 hours. In contrast, the average proliferation ratios of CsA-treated cells exhibited a time-dependent increase, with values of 1.05-fold at 12 hours (data not shown), 1.29-fold at 24 hours and 1.42-fold at 48 hours compared to the 0-hour baseline. The doubling times for untreated cells and CsA-treated cells were approximately 10,000 hours and 215 hours, respectively. Also, we detected the cell cycle of gingival fibroblasts exposed to CsA using flow cytometry analysis, and the results showed that CsA decreased the proportion of G_1_ phase cells while increasing the proportion of S and G_2_/M phase cells. Treatment with CsA resulted in reduced G_1_ cell cycle arrest and thus might cause gingival overgrowth. Several previous studies have demonstrated the effects of CsA on cell proliferation and the cell cycle using the following cells such as human hepatocellular carcinoma-derived cell line (HepG2 cells), human non-small cell lung cancer cell line (A549 cells), human umbilical vein endothelial cells (HUVECs), renal tubular epithelial cell line derived from distal tubular segment of nephron (Madin-Darby canine kidney (MDCK) cells). In HepG2 cells, CsA activated the ERK1/2 (extracellular signal-regulated kinase) signaling pathway, as a result, cell proliferation was activated [25]. Cell proliferation in A549 cells [26] and in HUVECs [27] was promoted by CsA treatment. The cell cycle transition to S and G_2_/M phases from G_1_ phase was enhanced by CsA in MDCK cells [28]. Also, in primary cultures of rat hepatocytes, CsA increased the relative number of S and G_2_/M cell cycle phase cells, as a result, cell proliferation was promoted [29]. CsA treatment enhanced cell cycle progression to G_2_/M from G_0_/G_1_ and S phases in gingival fibroblasts obtained from patients experiencing overgrowth in gingiva [30]. Our results in this study used human gingival fibroblasts that are similar to these previous studies and provide new observations in addition to these findings.

The machinery of cell cycle drives division of the cells. Cell cycle system also provides an opportunity to repair the damage of cells and to stop the transmission of defects to daughter cells [31]. Proteins called cyclins are key components of the cell cycle [32–36]. Cyclins bind and activate the cyclin-dependent kinases (CDKs, cyclins-associated catalytic partners), and provide substrate specificity to CDKs [32–36]. Cell cycle is divided into four phases: gap 1 (G_1_) phase (preparatory stage for DNA synthesis), DNA synthesis (S) phase, gap 2 (G_2_) phase (preparatory stage for mitosis) and mitosis (M) phase [32–36]. When cells traverse the G_1_ phase, the cells select one mechanism to progress depending on the mitogenic environment: a mechanism to activate a cell division program or a mechanism that enters a quiescent G_0_ state [32–36].

At the molecular level, when growth-promoting factors stimulate the cells, upregulation of the D-type cyclins (D1, D2 and D3) is induced, which CDK4 and CDK6 are activated in the cells [32–37]. This drives the cellular shift to the S phase from the G_1_ phase of the cell cycle, and leads to DNA synthesis [32–37]. CYCLIN D-activated CDK4/CDK6 then phosphorylates the retinoblastoma protein RB1 [38]. Ordinarily, the function of RB1 is to bind the E2F transcription factors to sequester the activity, thereby restraining entry into the S phase [38]. The release of E2F transcription factors is led by the RB1 phosphorylated by CYCLIN D-CDK4/CDK6 complexes, which the transcription of CYCLIN E1 and CYCLIN E2 (the target genes of E2F) is increased [38–40]. E-type cyclins then lead the hyperphosphorylation of RB1 via binding and activating to CDK2 [38–40]. E-type cyclins also lead the phosphorylation of numerous other proteins, which collectively regulate an irreversible commitment to S phase [38–40]. The KIP/CIP family of cell cycle inhibitors (P21^CIP1^, P27^KIP1^ and P57^KIP2^) directly bind to the complex of CYCLIN E-CDK2 and inhibit its catalytic activity [41]. CYCLIN E-CDK2 complex marks P27^KIP1^ for degradation via phosphorylation of it, which more progresses S phase of the cell cycle [41].

Throughout interphase, a cell cycle arrest is elicited when various factors impair DNA in the cells [16]. This phase grants permission to repair pathways to operate prior to commission to subsequent phases of the cell cycle [16]. In the G_1_ phase, P21 and P27 induce G_1_ cell cycle arrest via inhibition of CYCLIN D-CDK4/CDK6 and CYCLIN E-CDK2 complexes [41]. ATM (ataxia-telangiectasia mutated) and ATR (ataxia-telangiectasia mutated and rad3 related) activate P53, and then active P53 promotes P21 [42]. Additionally, SMAD3 and SMAD4 mediate the promotion of P21. MYC depresses the actions of P21 and P27 and leads the induction of CDC25A (activator of CDK2), and subsequently permits the hyperactivation of CYCLIN E-CDK complexes [43].

**Fig 5:**
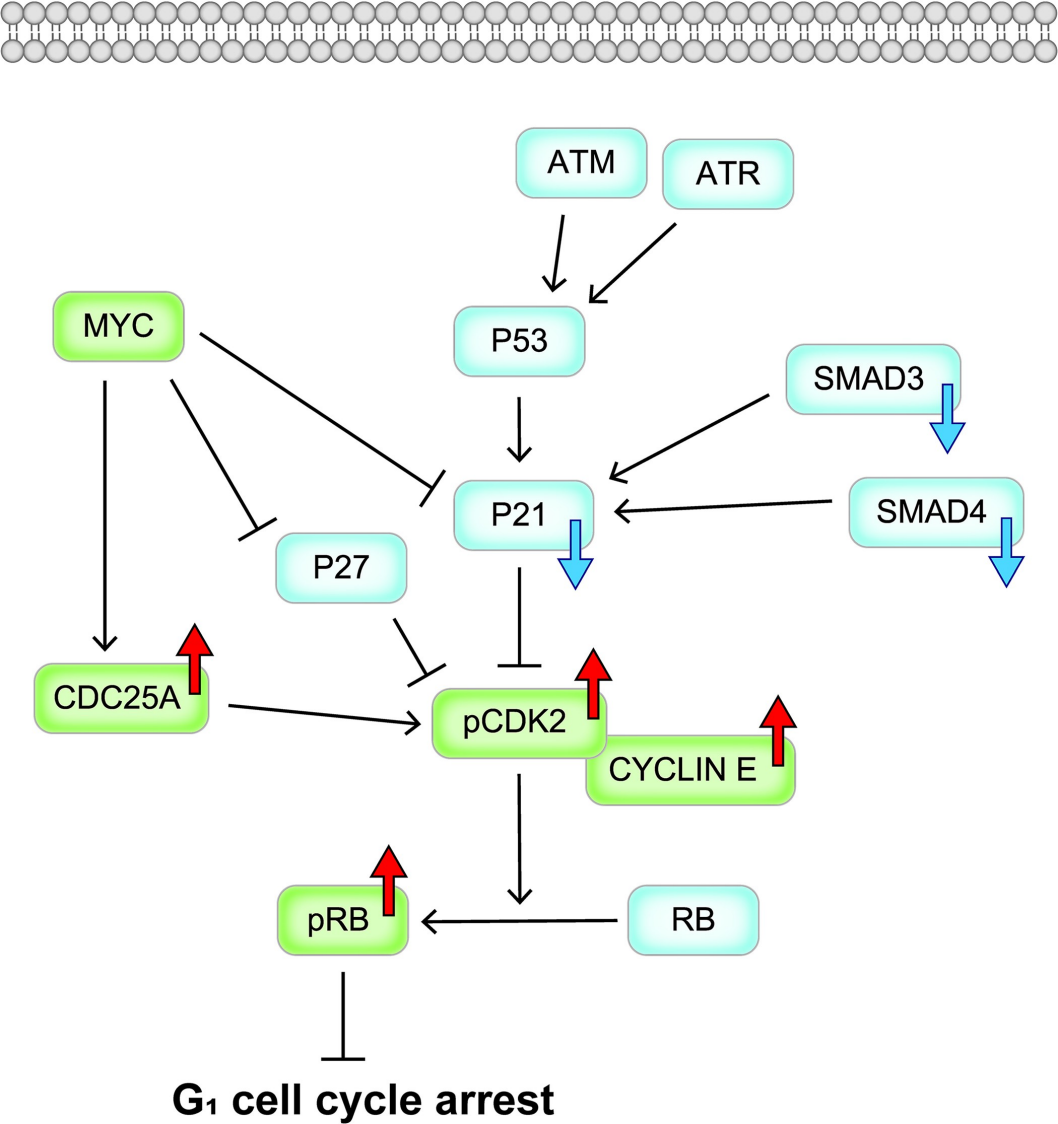
Schematic representation of G_1_ cell cycle arrest inhibited by CsA in gingival fibroblasts. CsA induces the downregulation of SMAD3 and SMAD4, which leads to a decrease in P21. CsA also induces the upregulation of CDC25A, CYCLIN E, phosphorylated CDK2 and phosphorylated RB, which results in the inhibition of G_1_ cell cycle arrest. Light blue (factors of G_1_ arrest-inducing signaling) and light green (factors of G_1_/S transition-inducing signaling) rectangles similarly denote the molecules analyzed in this study. The blue or red large arrows denote downregulation or upregulation, respectively, following CsA treatment. ATM, ATM serine/threonine kinase; ATR, ATR serine/threonine kinase; CDC25A, cell division cycle 25A; CYCLIN E, E-type cyclin; MYC, MYC proto-oncogene, bHLH transcription factor; pCDK2, phosphorylated cyclin dependent kinase 2; pRB1, phosphorylated RB transcriptional corepressor 1; P21, cyclin dependent kinase inhibitor 1A; P27, cyclin dependent kinase inhibitor 1B; P53, tumor protein p53; RB1, RB transcriptional corepressor 1; SMAD3, SMAD family member 3; SMAD4, SMAD family member 4.

To investigate the effects of CsA on the regulators of G_1_ cell cycle arrest, we investigated the mRNA expression levels of genes that include inducers (*CDC25A*, *CDK2*, *CYCLIN E1* and *MYC*) and inhibitors (*ATM*, *ATR*, *P21*, *P27*, *P53*, *RB1*, *SMAD3* and *SMAD4*) of the G_1_/S transition, and also examined the protein expression levels of CDC25A, CYCLIN E1, phospho-CDK2, phospho-RB1, P21, SMAD3 and SMAD4. We found that treatment with CsA enhanced the mRNA expression levels of *CDC25A* and *CYCLIN E1*, reduced the mRNA expression levels of *P21*, *SMAD3* and *SMAD4*, increased the protein expression levels of CDC25A, CYCLIN E, pCDK2 and pRB, and decreased the protein expression levels of P21, SMAD3 and SMAD4 in gingival fibroblasts. Based on those results, CsA may cause gingival overgrowth in gingival fibroblasts by the following mechanism: CsA increases phospho-CDK2 and CYCLIN E1 by upregulating CDC25A and downregulating P21 with the downregulation of SMAD3 and SMAD4, which results in the inhibition of G_1_ cell cycle arrest (shown schematically in Fig 5).

The mRNA expression levels of *CDK2* and *RB1* were found to be non-significantly down-regulated by CsA. In contrast, the protein expression levels of phospho-CDK2 and phospho-RB1 showed a significant up-regulation in response to CsA treatment. This suggests that CsA may either decrease or have no effect on the protein levels of CDK2 and RB1, while simultaneously enhancing the phosphorylation of these proteins. Previous research has indicated that post-transcriptional regulatory mechanisms—such as splicing, capping, polyadenylation, mRNA stability, translation regulation, and mRNA localization—play a more substantial role in influencing absolute protein levels compared to changes at the mRNA level [44]. Additionally, several critical processes contribute to determining protein expression levels beyond mere transcript concentration, including translation rates, modulation of translation rates, adjustments to a protein’s half-life, delays in protein synthesis, and protein transport [45]. Notably, the half-life of phospho-RB1 is regulated by degradation processes that involve ubiquitination [46].

Several studies previously demonstrated the effects of CsA on cell cycle regulators using several types of mammalian cells. CsA induced the upregulation of RB1 phosphorylation after enhancing the expression of CDK4, CYCLIN D1 and RB proteins in human gingival fibroblasts [47]. The increase in growth of A431 human epidermoid carcinoma (CRL-2592) cells treated with CsA was associated with enhancement of the expression of CYCLIN D1 protein [48]. The mRNA expression level of CYCLIN B1 was increased in human gingival fibroblasts obtained from patients who had gingival overgrowth caused by CsA compared with normal gingival fibroblasts obtained from healthy individuals [30]. CsA increased the mRNA and protein expression levels of CYCLIN D1, decreased the mRNA expression level of P27, and upregulated the protein expression levels of phospho-AKT/AKT, which overcame cell cycle arrest in the G_1_ and G_2_ phases following the decreased protein expression of P27 in A549 cells [26]. Also, the protein expression levels of CYCLIN D1 and CYCLIN E1 as well as PCNA (proliferating cell nuclear antigen) increased but without any change in P27 levels in primary cultures of rat hepatocytes treated with CsA [29]. In addition to those findings, our results not only clarify the mechanism of CsA-induced gingival overgrowth but also offer new insights concerning the effects of CsA on the cell cycle in human gingival fibroblasts.

Ink4 family members (P16^Ink4a^, P15^Ink4b^, P18^Ink4c^ and P19^Ink4d^) impair the activities of CYCLIN D-CDK4/6 complexes and result in the G_1_ cell cycle arrest [49]. Similarly, P21 and P27 regulate the inhibition of CYCLIN E-CDK2 complex and subsequently induce the G_1_ cell cycle arrest [49]. When TGF-β binds its receptor, SMAD2/3 and SMAD4 are activated, and the transcriptional activity of P15 is promoted [50]. CYCLIN D1 degradation is regulated by glycogen synthase kinase-3β (GSK-3β) and dual-specificity tyrosine-phosphorylation regulated kinase 1B (DYRK1B) [51]. Also, P21 expression is mediated under versatile and strict regulatory control by many regulators such as breast cancer susceptibility gene 1 (BRCA1), double homeobox 4 (Dux4), Promyelotic zinc finger (PLZF), etc [52]. Thus, future studies should aim to clarify the effects of CsA on these factors in gingival fibroblasts.

## Conclusion

CsA may cause gingival overgrowth in gingival fibroblasts by the following mechanism: CsA increases phospho-CDK2 and CYCLIN E1 by upregulating CDC25A and downregulating P21 with the downregulation of SMAD3 and SMAD4, which results in the inhibition of G_1_ cell cycle arrest.

## Supporting information

S1 Fig Original images for explaining Fig 4. (TIF)

S1 Data Supplementary data for Fig 1. (PDF)

S2 Data Supplementary data for Fig 2. (PDF)

S3 Data Supplementary data for Fig 3. (PDF)

S4 Data Supplementary data for Fig 4. (PDF)

## Abbreviations

ATM: ATM serine/threonine kinase
ATR: ATR serine/threonine kinase
CDC25A: cell division cycle 25A
CDK2: cyclin dependent kinase 2
CsA: cyclosporine A
CYCLIN E1: E-type cyclin
MYC: MYC proto-oncogene
bHLH: transcription factor
P21: cyclin dependent kinase inhibitor 1A
P27: cyclin dependent kinase inhibitor 1B
P53: tumor protein p53
RB1: RB transcriptional corepressor 1
SMAD3: SMAD family member 3
SMAD4: SMAD family member 4
GAPDH: glyceraldehyde-3-phosphate dehydrogenase
