# Supplementary figures and images for: Cyclosporine A causes gingival overgrowth via reduced G1 cell cycle arrest in gingival fibroblasts
Source: PLoS One. 2024 Dec 20;19(12):e0309189. doi: 10.1371/journal.pone.0309189 (PMC11661605; doi:10.1371/journal.pone.0309189)

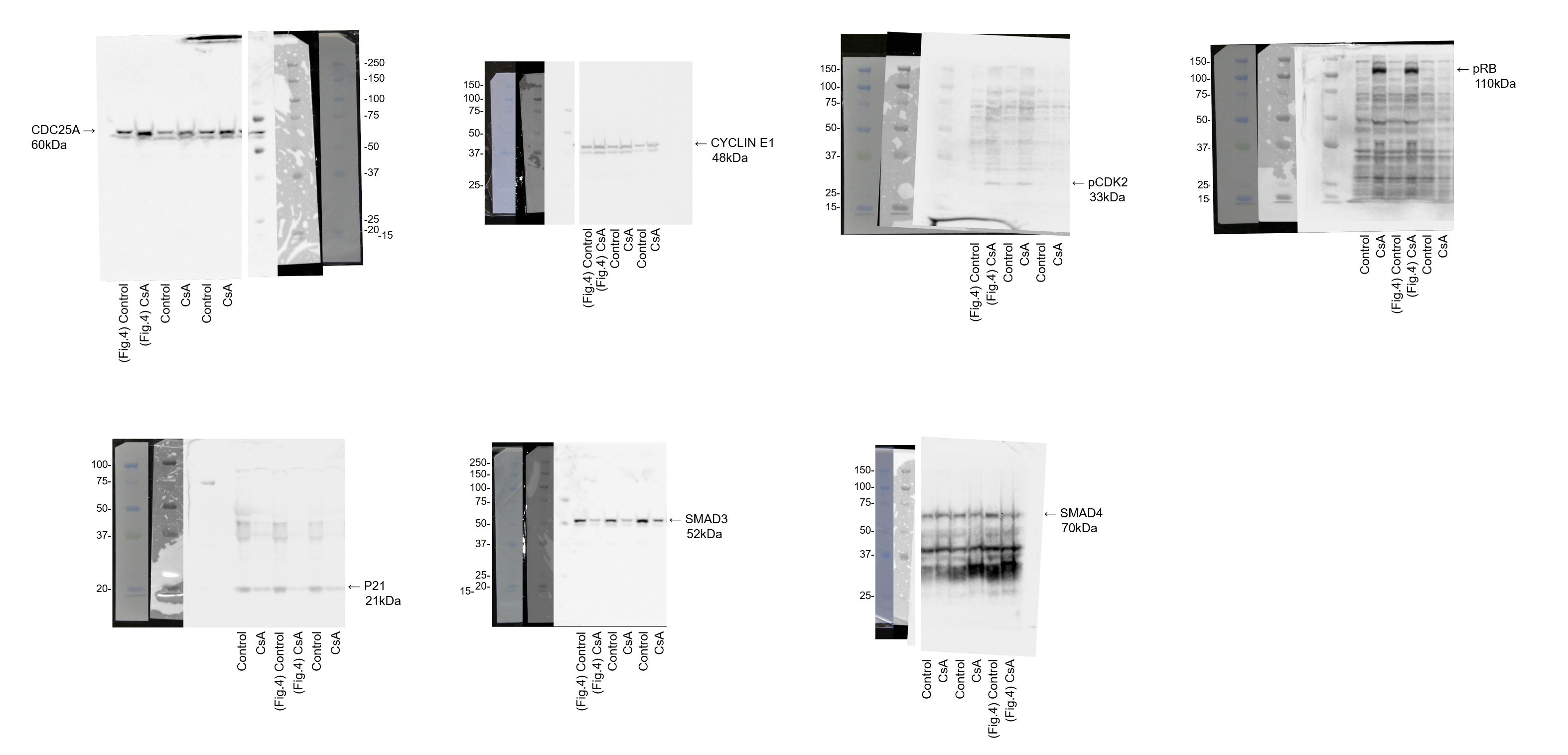

Supplement: S1 Fig — (TIF) [file pone.0309189.s001.tif]
