# Supplementary material for: Cyclosporine A causes gingival overgrowth via reduced G1 cell cycle arrest in gingival fibroblasts
Source: PLoS One. 2024 Dec 20;19(12):e0309189. doi: 10.1371/journal.pone.0309189 (PMC11661605; doi:10.1371/journal.pone.0309189)
Supplement: S1 Data — (PDF) [file pone.0309189.s002.pdf]

S1 Data

		DATA	DATA	DATA	DATA	**Average**	**SEM**
0h	Control	1.0	1.0	1.0	1.0	**1.0**	**0**
0h	Cs A	1.0	1.0	1.0	1.0	**1.0**	**0**
24h	Control	1.13	1.23	1.20	1.08	**1.16**	**0.029**
24h	Cs A	1.37	1.29	1.20	1.28	**1.29**	**0.030**
48h	Control	1.20	0.84	0.95	1.02	**1.00**	**0.066**
48h	Cs A	1.45	1.37	1.66	1.20	**1.42**	**0.083**
