# Supplementary material for: Cyclosporine A causes gingival overgrowth via reduced G1 cell cycle arrest in gingival fibroblasts
Source: PLoS One. 2024 Dec 20;19(12):e0309189. doi: 10.1371/journal.pone.0309189 (PMC11661605; doi:10.1371/journal.pone.0309189)
Supplement: S2 Data — (PDF) [file pone.0309189.s003.pdf]

S2 Data

		G0/G1	S	G2/M
Control	DATA	93.6	2.4	4.0
Control	DATA	94.9	1.2	3.9
Control	DATA	94.9	1.4	3.7
Control	DATA	95.2	0.9	3.9
**Control**	**Average**	**94.7**	**1.5**	**3.9**
**Control**	**SEM**	**0.3**	**0.3**	**0.1**
Cs A	DATA	65.6	16.0	18.4
Cs A	DATA	81.0	9.1	9.9
Cs A	DATA	83.7	7.5	8.8
Cs A	DATA	81.2	8.3	10.5
**Cs A**	**Average**	**77.9**	**10.2**	**11.9**
**Cs A**	**SEM**	**4.1**	**2.0**	**2.2**
