# Supplementary material for: Cyclosporine A causes gingival overgrowth via reduced G1 cell cycle arrest in gingival fibroblasts
Source: PLoS One. 2024 Dec 20;19(12):e0309189. doi: 10.1371/journal.pone.0309189 (PMC11661605; doi:10.1371/journal.pone.0309189)
Supplement: S3 Data — (PDF) [file pone.0309189.s004.pdf]

S3 Data

	CDC25A	CDC25A	CDK2	CDK2	CYCLIN E1	CYCLIN E1	MYC	MYC
	Control	Cs A	Control	Cs A	Control	Cs A	Control	Cs A
DATA	1.12	1.51	1.43	0.93	0.85	1.35	1.75	2.80
DATA	1.00	1.98	0.92	0.80	1.18	1.67	0.62	0.72
DATA	0.89	1.72	0.76	0.80	1.09	1.80	0.62	0.50
**Average**	**1.00**	**1.74**	**1.04**	**0.84**	**1.04**	**1.61**	**1.00**	**1.34**
**SEM**	**0.07**	**0.14**	**0.20**	**0.04**	**0.10**	**0.14**	**0.38**	**0.73**

	ATM	ATM	ATR	ATR	P21	P21	P27	P27
	Control	Cs A	Control	Cs A	Control	Cs A	Control	Cs A
DATA	1.55	1.23	1.45	0.88	0.87	0.39	1.08	0.93
DATA	0.74	1.03	0.82	0.82	1.30	0.20	1.48	0.64
DATA	0.88	1.03	0.84	0.86	0.87	0.23	0.63	0.49
**Average**	**1.05**	**1.09**	**1.04**	**0.85**	**1.01**	**0.27**	**1.06**	**0.69**
**SEM**	**0.25**	**0.07**	**0.21**	**0.02**	**0.14**	**0.06**	**0.24**	**0.13**

	P53	P53	RB1	RB1	SMAD3	SMAD3	SMAD4	SMAD4
	Control	Cs A	Control	Cs A	Control	Cs A	Control	Cs A
DATA	1.35	1.10	1.46	0.97	1.36	0.30	1.28	0.35
DATA	0.93	0.79	0.91	0.85	0.91	0.25	1.02	0.26
DATA	0.80	0.88	0.75	0.87	0.81	0.29	0.76	0.29
**Average**	**1.03**	**0.92**	**1.04**	**0.90**	**1.03**	**0.28**	**1.02**	**0.30**
**SEM**	**0.17**	**0.09**	**0.22**	**0.04**	**0.17**	**0.01**	**0.15**	**0.03**
