# Supplementary material for: Cyclosporine A causes gingival overgrowth via reduced G1 cell cycle arrest in gingival fibroblasts
Source: PLoS One. 2024 Dec 20;19(12):e0309189. doi: 10.1371/journal.pone.0309189 (PMC11661605; doi:10.1371/journal.pone.0309189)
Supplement: S4 Data — (PDF) [file pone.0309189.s005.pdf]

S4 Data

	CDC25A	CDC25A	CYCLIN E1	CYCLIN E1	pCDK2	pCDK2	pRB	pRB
	Control	Cs A	Control	Cs A	Control	Cs A	Control	Cs A
DATA	1.0	1.40	1.0	1.70	1.0	3.45	1.0	3.71
DATA	1.0	1.87	1.0	1.76	1.0	2.63	1.0	2.97
DATA	1.0	1.54	1.0	1.98	1.0	3.35	1.0	3.68
**Average**	**1.00**	**1.60**	**1.00**	**1.81**	**1.00**	**3.14**	**1.00**	**3.45**
**SEM**	**0.00**	**0.14**	**0.00**	**0.09**	**0.00**	**0.26**	**0.00**	**0.24**

	P21	P21	SMAD3	SMAD3	SMAD4	SMAD4
	Control	Cs A	Control	Cs A	Control	Cs A
DATA	1.0	0.65	1.0	0.46	1.0	0.71
DATA	1.0	0.64	1.0	0.49	1.0	0.57
DATA	1.0	0.66	1.0	0.39	1.0	0.34
**Average**	**1.00**	**0.65**	**1.00**	**0.44**	**1.00**	**0.54**
**SEM**	**0.00**	**0.00**	**0.00**	**0.03**	**0.00**	**0.11**
